# Bats partition activity in space and time in a large, heterogeneous landscape

**DOI:** 10.1002/ece3.7504

**Published:** 2021-05-01

**Authors:** Elizabeth A. Beilke, Rachel V. Blakey, Joy M. O’Keefe

**Affiliations:** ^1^ Department of Natural Resources and Environmental Sciences University of Illinois at Urbana‐Champaign Urbana IL USA; ^2^ Center for Bat Research, Outreach, and Conservation Indiana State University Terre Haute IN USA; ^3^ La Kretz Center for California Conservation Science Institute of the Environment and Sustainability University of California Los Angeles CA USA; ^4^ Department of Ecology and Evolutionary Biology University of California Los Angeles CA USA

**Keywords:** activity patterns, bats, habitat use, interspecific competition, resource partitioning, temporal partitioning

## Abstract

Diverse species assemblages theoretically partition along multiple resource axes to maintain niche separation between all species. Temporal partitioning has received less attention than spatial or dietary partitioning but may facilitate niche separation when species overlap along other resource axes. We conducted a broad‐scale acoustic study of the diverse and heterogeneous Great Smoky Mountains National Park in the Appalachian Mountains. Between 2015 and 2016, we deployed acoustic bat detectors at 50 sites (for a total of 322 survey nights). We examined spatiotemporal patterns of bat activity (by phonic group: Low, Mid, and *Myotis*) to test the hypothesis that bats partition both space and time. *Myotis* and Low bats were the most spatially and temporally dissimilar, while Mid bats were more general in their resource use. Low bats were active in early successional openings or low‐elevation forests, near water, and early in the evening. Mid bats were similarly active in all land cover classes, regardless of distance from water, throughout the night. *Myotis* avoided early successional openings and were active in forested land cover classes, near water, and throughout the night. *Myotis* and Mid bats did not alter their spatial activity patterns from 2015 to 2016, while Low bats did. We observed disparate temporal activity peaks between phonic groups that varied between years and by land cover class. The temporal separation between phonic groups relaxed from 2015 to 2016, possibly related to changes in the relative abundance of bats or changes in insect abundance or diversity. Temporal separation was more pronounced in the land cover classes that saw greater overall bat activity. These findings support the hypothesis that niche separation in diverse assemblages may occur along multiple resource axes and adds to the growing body of evidence that bats partition their temporal activity.

## Graphical abstract

We describe the patterns of spatiotemporal partitioning exhibited by bats in the Southern Appalachians. We provide evidence for both spatial and temporal partitioning in this bat assemblage and demonstrate that temporal activity patterns vary by bat phonic group, year, and land cover class.

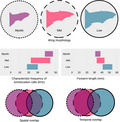

## 1 INTRODUCTION

Diverse species assemblages theoretically partition along an indeterminate number of resource axes, but the nature and relative importance of these axes remain a hot topic in ecology. We typically study resource partitioning along three dominant resource axes: space, diet, and time. Assemblages may partition along these axes simultaneously (Hearn et al., 2018; Luiselli, 2006; Wilson, 2010), and more diverse assemblages should partition along multiple axes to maintain niche separation among all species. Temporal partitioning has received less attention than spatial and dietary partitioning and relatively few studies have attempted to understand species interactions across both time and space (Frey et al., 2017; Kronfeld‐Schor & Dayan, 2003).

Insectivorous bat assemblages are diverse and partition by space and dietary preference. Approximately 70% of the >1,400 bat species worldwide are insectivorous (Burgin et al., 2018), with most local assemblages comprising several different species. Spatial and dietary partitioning are thought to be the most prevalent form of resource partitioning in bats because their ecomorphology dictates their flight patterns and, therefore, habitat use and feeding ecology (Aldridge & Rautenbach, 1987; Neuweiler, 1984; Norberg & Rayner, 1987). Previous work has demonstrated sympatric bat species partition space horizontally—at coarse (Arlettaz, 1999; Nicholls & Racey, 2006) and fine scales (Saunders & Barclay, 1992)—and vertically (Kalcounis et al., 1999; Menzel, Menzel, et al., 2005; Müller et al., 2013). Sympatric species also partition prey by size (Dodd et al., 2015), behavior (Mata et al., 2018), and taxa (Cravens et al., 2018; Whitaker, 2004), although spatial and dietary partitioning need not be mutually exclusive (Roswag et al., 2015; Saunders & Barclay, 1992).

Given our theoretical understanding of temporal partitioning, we should expect bats to partition time; however, few studies examine temporal partitioning in bats and the results sometimes conflict. Temporal partitioning facilitates niche separation among sympatric predators, particularly where prey exhibit peaks of activity (Ramesh et al., 2012). Because insect activity is periodic within a night (Lewis & Taylor, 1965; Rydell et al., 1996), insectivorous bats are well‐situated for temporal resource partitioning. Several studies have detected temporal partitioning in bats (e.g., Kunz, 1973), a phenomenon intensified by spatial overlap (Emrich et al., 2014; Mancina et al., 2012) or resource scarcity. For example, bats temporally partition pond visitation times in water‐scarce environments (Adams & Thibault, 2006; Lambert et al., 2018; Razgour et al., 2011). Temporal partitioning may be particularly important to bats that are biomechanically constrained from expanding their fundamental spatial niche (Mayberry et al., 2020). However, bats do not always exhibit clear patterns of temporal partitioning, and it may not be an important mechanism of niche separation where bats exhibit strong patterns of spatial separation (Arlettaz, 1999; Fenton & Rautenbach, 1987).

Under ideal conditions, temporal partitioning may be a strategy of flexibility rather than necessity, employed by species occupying similar spatial or dietary niches. For example, *Lasionycteris noctivagans* expands its temporal niche in the presence of *Eptesicus fuscus* (Reith, 1980), a species with similar ecomorphology (Norberg & Rayner, 1987) and diet (Whitaker, 2004), and *Myotis sodalis* shifts its foraging activity to earlier in the evening in the presence of the ecologically similar *M. septentrionalis* (Lee & McCracken, 2004). If temporal partitioning is a flexible strategy, its presence may not be apparent in small datasets, especially if we sample a subset of species assemblages at a few similar sites. Moreover, temporal partitioning may not always be a viable strategy for predators, as it relies on the temporal separation of their prey. It stands to reason that heterogeneous landscapes that support a diverse insect assemblage should provide greater opportunity for bats to partition temporally; however, this has not been formally tested, thus emphasizing the importance of scope and heterogeneity in temporal partitioning studies.

Large‐scale disturbances can influence temporal partitioning in diverse ways. For example, understory‐foraging frugivorous bats alter their temporal activity patterns in response to reduced‐impact logging (Castro‐Arellano et al., 2009). Likewise, changes in bat or insect assemblages could prompt bats to modify their spatiotemporal strategies. Recent evidence suggests white‐nose syndrome (WNS), a fungal disease that has caused declines in several *Myotis* species, has caused shifts in resource use by bats. For example, there is evidence that the spatiotemporal (Jachowski et al., 2014; Teets, 2018) niches of bats unaffected by WNS have relaxed in WNS‐positive landscapes, presumably due to the decline of *Myotis* in those assemblages. These findings support the hypothesis that bats may exhibit flexibility in their spatiotemporal niches. However, neither of those studies examined the fine‐scale patterns of temporal partitioning that occur across years and land cover classes.

We examined spatiotemporal partitioning in bat activity across a large area with a significant elevation gradient in the ecologically diverse Appalachian Mountains. We evaluated the relationship between land cover class, distance to water, and phonic group activity to assess spatial partitioning and quantified the degree of temporal overlap between phonic group pairs. We also investigated the consistency of patterns of spatiotemporal activity across the 2‐year study period and examined how temporal partitioning varied with land cover class. We hypothesized that bats would partition time and space in this diverse landscape. Specifically, we predicted that *Myotis* and Low bats would be the most spatially and temporally separated given their distinct ecomorphology. Mid bats, with intermediate ecomorphology, should be more generalist in their spatiotemporal resource use. During this study, populations of four bat species in our area were in decline due to the effects of WNS (O'Keefe et al., 2019). We predicted that we would observe changes in spatiotemporal resource use from 2015 to 2016, particularly along the temporal axis, due to decreased bat abundance and, therefore, competition. We predicted that phonic group activity patterns would be the most disparate in land cover classes with greater overall bat activity, given temporal partitioning is likely to increase with spatial overlap.

## 2 MATERIALS AND METHODS

### 2.1 Study area

**FIGURE 1:**
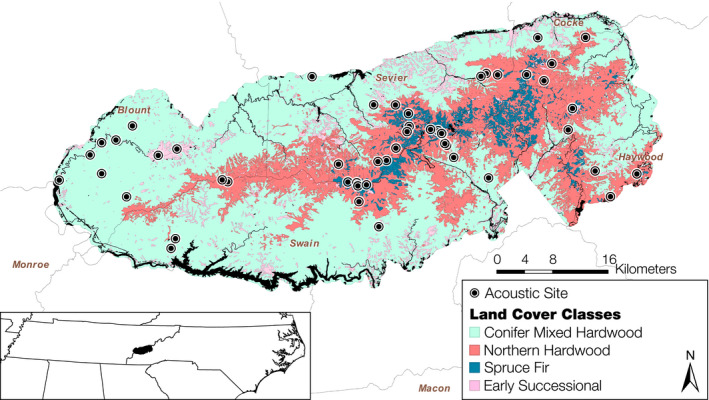
Bats were sampled acoustically at 50 sites (ringed circles) within five counties in Great Smoky Mountains National Park in the Appalachian Mountains on the border of Tennessee and North Carolina, USA (see insert). Map shows dominant land cover classes: conifer‐mixed hardwood (light green), northern hardwood (red), spruce‐fir (blue), and early successional (pink). The black linear features represent sources of water within the park boundary

We conducted this study in Great Smoky Mountains National Park (GRSM; 35°36′42″N, 83°29′22″W, Figure 1), a 211,000 ha protected area set in the Appalachian Mountains of the eastern United States, during the summers of 2015 and 2016. Approximately 97% of the park is vegetated and elevation ranges from 259 to 2,026 m. Vegetation cover in the park can be separated into three major classes broadly associated with elevation: low‐elevation conifer‐mixed hardwood forest (CM; 64%), mid‐elevation northern hardwood forest (NH; 24%), and high‐elevation spruce‐fir forest (SF; 3%). The park additionally contains a variety of early successional openings (ES; 6%) unrelated to elevation, both natural and anthropogenic in origin. Climate also varies with topography. From May to August of 2015 and 2016, the high peaks received more rain (59 and 74 cm, respectively) than the lowlands (50 and 44 cm, respectively) and were 9°C cooler and 8%–14% more humid (NPS, 2019).

### 2.2 Local Bat Fauna

Thirteen species of bat have been documented in the park including the northern long‐eared bat (*Myotis septentrionalis*), Indiana bat (*Myotis sodalis*), small‐footed bat (*Myotis leibii*), eastern red bat (*Lasiurus borealis*), hoary bat (*Lasiurus cinereus*), silver‐haired bat (*Lasionycteris noctivagans*), tricolored bat (*Perimyotis subflavus*), evening bat (*Nycticeius humeralis*), Rafinesque's big‐eared bat (*Corynorhinus rafinesquii*), Seminole bat (*Lasiurus seminolus*), gray bat (*Myotis grisescens*), little brown bat (*Myotis lucifugus*), and big brown bat (*Eptesicus fuscus*); it is also possible that the Mexican free‐tailed bat (*Tadarida brasiliensis*) occurs there (Table 1; Carpenter, 2017; O'Keefe et al., 2019). The arrival of WNS in the park in 2009 has led to dramatic declines in capture rates for *M. septentrionalis*, *M. sodalis*, *M. lucifugus*, and *P. subflavus*—capture rates for these species were 82%–99% lower in 2014–2016 than in 2009–2012 (O'Keefe et al., 2019). From 2015 to 2016, *E. fuscus*, *L. borealis*, *L. noctivagans*, and *Myotis leibii* comprised the majority of bats captured in the park (Table 1; Carpenter, 2017; O'Keefe et al., 2019).

**TABLE 1:** Summary of bat phonic groups used in our study: species composition, their relative capture rate, ecomorphological indices (Norberg & Rayner, 1987), and foraging strategy

Phonic group	Common name	Scientific name	Capture rate (%)	Avg mass (g)	Wing aspect ratio	Wing loading	Foraging strategy
*Myotis*	Eastern small‐footed bat	*Myotis leibii*	5.8	5.0	6.1	6.7	Narrow‐space, hawking foragers
	Indiana bat	*Myotis sodalis*	1.4	7.4	5.4	6.5	Narrow‐space, hawking foragers
	Northern long‐eared bat	*Myotis septentrionalis*	1.0	7.0	5.8	6.8	Narrow‐space, hawking‐gleaning foragers
	Little brown bat	*Myotis lucifugus*	0.8	7.9	6	7.5	Narrow‐space, hawking‐trawling foragers
	Gray bat	*Myotis grisescens*	0.4	12.1	6.4	8.2	Edge‐space, hawking‐trawling foragers
Mid	Eastern red bat	*Lasiurus borealis*	25.3	11.2	6.7	14	Edge‐space, hawking foragers
	Tricolored bat	*Perimyotis subflavus*	4.4	5.6	6.2	5.6	Edge‐space, hawking foragers
	Rafinesque's big‐eared bat	*Corynorhinus rafinesquii*	3.6	10.0	5.9	5.9	Narrow‐space, gleaning foragers
	Evening bat	*Nycticeius humeralis*	2.5	9.5	6.8	10.7	Edge‐space, hawking foragers
	Seminole bat	*Lasiurus seminolus*	0.0	10.9	–	–	Edge‐space, hawking foragers
Low	Big brown bat	*Eptesicus fuscus*	46.6	18.6	6.4	9.4	Open‐space, hawking foragers
	Silver‐haired bat	*Lasionycteris noctivagans*	6.7	10.6	6.6	8.2	Open‐space, hawking foragers
	Hoary bat	*Lasiurus cinereus*	1.5	25.7	8.1	16.5	Open‐space, hawking foragers
	Mexican free‐tailed bat	*Tadarida brasiliensis*	0.0	12.5	8.2	11.5	Open‐space, hawking foragers

Note

Foraging strategy is informed by Norberg and Rayner (1987) and Denzinger and Schnitzler (2013). Capture rate is based on 841 captures from 2015 to 2016 (O’Keefe et al., 2019, Carpenter, 2017). High aspect ratio (the square of the wingspan divided by wing area) is associated with long, narrow wings. High wing loading (weight divided by wing area) indicates a heavier body size relative to wing size. Slow, maneuverable flight is generally associated with low wing loading and aspect ratio. Fast, efficient, agile flight is generally associated with high wing loading and aspect ratio.

### 2.3 Survey design

We conducted a comprehensive, spatially distributed acoustic bat survey of GRSM. We selected our survey sites using the Generalized Random‐Tessellation Stratified method, as this method yields a spatially balanced, stratified design while allowing for substitution of unsuitable sites (Stevens & Olsen, 2004). The GRTS sampling was conducted using the *spsurvey* package within R and spatial data processing was undertaken in QGIS v2.8 (QGIS Development Team, 2015). We plotted 50 potential sites, with 50 oversample sites, each of which belonged to 1 of 8 categories, defined by both land cover class (ES, CM, NH, and SF; Table 2 for descriptions) and proximity to permanent sources of water (third order and larger streams; near: <1,000 m from a stream or river; far: >1,000 m from a stream or river), although we treated distance from water as a continuous variable in our analysis. We constrained water sources this way because bats are more likely to use larger, calmer sources of water relative to smaller, faster‐flowing sources of water for drinking and foraging (Razgour et al., 2010; Seidman & Zabel, 2001; von Frenckell & Barclay, 2011). We measured site characteristics with a laser range finder and densiometer and characterized vegetation using digital GRSM National Parks vegetation maps (Madden et al., 2004; Table 2). We used the detailed vegetation data to assign each stand to one of the four land cover classes described above: CM, NH, SF, or ES. We measured proximity to permanent water features (all linear) in the National Hydrography Dataset NHD Plus v2 (McKay et al., 2012). We ground‐truthed all land cover class assignments and used the oversample to make substitutions when necessary (e.g., if site access was too dangerous), to achieve a total of 50 sites. Our dataset included some sites near each other, as eliminating these would have violated the GRTS method. The closest sites were 102 m apart, while the farthest were 83 km apart. Overall, spatial auto‐correlation in nightly bat activity was low (*Moran's‐I* = 0.05, *p* = .57).

**TABLE 2:** Dominant land cover classes in Great Smoky Mountains and some environmental characteristics of sites sampled

Land cover class	Included land cover types (Madden et al., 2004)	Predicted degree of clutter	Elevation (m)	Live stem count	Canopy closure (%)	Basal area (m^2^ per ha/10)	Trail ht. (m)	Trail or gap width (m)	Avg. understory ht. (m)	Avg. nightly temp. (°C)
Conifer‐mixed hardwood	Chestnut oak forest, floodplain forest, hemlock forest, montane cove forest, montane oak‐hickory forest, white pine forest, yellow pine forest	Medium	917 ± 57	39 ± 6	64 ± 7	3.0 ± 0.4	19 ± 1	5 ± 1	0.8 ± 0.2	19 ± 0.4
Northern hardwood	High‐elevation beech/red oak forest, high‐elevation red oak/white oak forest, northern hardwood/acid hardwood forest, norther hardwood/boulder field forest	High	1,372 ± 45	42 ± 4	86 ± 3	3.4 ± 0.4	14 ± 1	6 ± 1	1.5 ± 1.3	16 ± 0.4
Spruce‐fir	Spruce‐fir forest	High	1736 ± 42	91 ± 25	53 ± 6	4.5 ± 0.7	13 ± 1	5 ± 1	0.9 ± 1.0	15 ± 0.8
Early successional	Ericaceous shrubs (heath bald type), ericaceous shrubs (nonheath bald type), grassy balds, successional or modified vegetation, tornado damage in 2011	Low	1,056 ± 151	18 ± 4	10 ± 5	1.2 ± 0.3	–	133 ± 57	1.4 ± 0.6	18 ± 0.8

Note

Characteristics were measured at each acoustic survey site with a GPS unit, laser range finder, densiometer, or weather station, where appropriate. All values are presented as means ± standard error.

### 2.4 Bat surveys

We recorded bat echolocation calls with a Pettersson D500X detector for 2–15 nights/site (6.55 ± 2.69, mean ± *SD*) from May to August in 2015 and 2016, for a total of 322 survey nights. At any given time, we surveyed 1–7 random sites simultaneously. Of the 50 sites, 39 were surveyed in both 2015 and 2016, 8 were surveyed only in 2015, and 3 only in 2016. We set detectors to a 500 kHz sampling rate, with pretrigger off, recording length = 0.3 (in 2015) or 5.0 (in 2016) sec, trigger‐sense = very high, gain = 45, and trigger level = 160.

We deployed detectors on hiking trails or in early successional openings to standardize probability of detection across land cover classes and made the following assumptions in our analyses: that all bat passes were independent, all phonic groups had an equal probability of detection, and that bats were distributed randomly in vertical space. These assumptions are congruent with many acoustic studies (see Sherwin et al., 2000). We attached each directional microphone to its detector using a 7.5 m‐cable, raised to a height of 3.0 m above the ground using PVC piping, and supported by a dowel rod extending 0.3 m away from the top of the post at a 5° decline from horizontal to prevent water from pooling on the surface of the microphone. We programmed our detectors to record from 30 min before sunset to 30 min after sunrise, according to data for a point in GRSM, 35°36′30″ North/83°56′09″ West and discarded any partial‐night data. We did not deploy detectors when there was a strong chance of rain in the forecast. We measured average nightly temperature and relative humidity for the duration of each deployment using Honest Observer by Onset Pro units deployed in a weatherproof case within 10 m of the acoustic detector and deployed at the same height of 3 m.

We identified acoustic files using the SonoBat 3.2.2 (Szewczak, 2013) automatic classifier at its default settings. To address the difficulty of assigning species to acoustic calls (see Russo et al., 2018), we re‐assigned all calls that were identified to the species or genus level to one of three phonic groups: Low, Mid, or *Myotis*. Phonic groups are closely related to clutter tolerance and foraging strategy in bats (see Table 1 for a summary). The Low group primarily contains open‐space aerial hawkers; the Mid group contains edge‐adapted aerial hawkers, and the *Myotis* group primarily contains narrow‐space aerial hawkers (Norberg & Rayner, 1987). Two bats do not fit these classifications: *Myotis grisescens*, an edge‐adapted, trawling‐hawking *Myotis*, and *Corynorhinus rafinesquii*, a gleaning Mid bat. It is unlikely this influenced our work, as *M. grisescens* is only rarely captured in the park (Table 1) and *C. rafinesquii* produce low‐intensity echolocation calls that are less likely to be recorded (Fenton, 1982). We defined bat activity as the number of nightly bat passes.

### 2.5 Statistical analysis

Before examining resource use, we used a Kruskal–Wallis test to determine whether there were significant differences between the mean number of bat calls recorded per site across years that might be attributed to the variable call file lengths we collected in 2015 and 2016 (bat activity ~ year). We detected no significant differences across years (*H* = 6.75e‐05, *df* = 1, *p* = .99) and concluded that call file length did not have a strong influence on the number of calls we recorded; thus, we treated data from 2015 and 2016 the same in our analyses.

To investigate patterns of spatial use by bats, we constructed a base generalized linear mixed effect model for each response variable (nightly Low, Mid, or *Myotis* passes) with site as a random intercept and the negative binomial family to account for overdispersion in the data. For each phonic group, the base model also contained land cover class and distance from water as predictor values and mean nightly temperature and relative humidity—which influence bat activity, atmospheric attenuation of acoustic calls, and probability of detection (Kaiser & O’Keefe, 2015; Lawrence & Simmons, 1982)—as covariates. Survey effort (number of nights/site) was not positively correlated to nightly bat activity (*R* = −.09), so we did not include it as a covariate in our models. We then used AICc to compare the base model with two models testing the effect of year on spatial use. One model contained year as an interactive effect with land cover class and distance from water, which tested the prediction that phonic groups changed their realized spatial niches from 2015 to 2016. The other model contained year as an additive effect, testing the prediction that phonic group activity levels changed from 2015 to 2016 but spatial activity patterns did not. The most parsimonious of the three models was then retained as the plausible model for that phonic group. To evaluate the importance of land cover class, which was a categorical variable, we compared the retained plausible model to a null model containing every variable in the plausible model except land cover class. To evaluate the importance of distance from water, a continuous variable, we evaluated the *z* scores in the plausible model summary. We assessed the fit of the plausible model by checking its residual plots and calculated confidence intervals around predicted relationships, incorporating fixed‐effects uncertainty only, using a parametric bootstrap approach with 1,000 resamples. All analyses were performed in Program R 3.6.3 (R Core Team, 2020). We used the *glmmTMB* package to fit mixed‐effects models, the *DHARMa* package to evaluate model fit, and the *lme4* and *boot* packages to calculate confidence intervals. We evaluated significance at *p* < .05 and express uncertainty in model predictions with 95% confidence intervals.

To examine the degree of temporal overlap in activity between phonic group pairs, we used a nonparametric kernel density estimation procedure (Linkie & Ridout, 2011; Ridout & Linkie, 2009). Following the methods outlined in Ridout and Linkie (2009), we converted the timestamp of each acoustic file to radians and used a kernel density estimation to generate a probability density distribution for phonic group pairs. We used the *overlap* package in Program R (Meredith & Ridout, 2014) to generate an overlap term from the mutual area under the two activity curves (Δ_4_), ranging from 0 (no activity overlap) to 1 (total activity overlap). We calculated 95% confidence intervals for all estimates from 1,000 bootstrap resamples, using them to determine whether the degree of overlap between phonic group pairs varied with year; if estimates did not overlap, we considered the degree of temporal overlap significantly different. We used the same nonparametric kernel density estimation procedure to explore temporal activity trends among the three phonic groups in different land cover classes. We quantified temporal overlap using the aforementioned methods but report the average Δ_4_ for each land cover class (calculated by averaging the overlap values for each of the three phonic group pairs). This method prevented us from calculating confidence intervals but made interpretation more succinct by eliminating 66 pairwise comparisons.

## 3 RESULTS

We recorded 19,502 bat passes; of these, 10690 were identifiable to species or species group. In 2015, 3,321 bat passes were classified as Low bat calls, 647 as Mid bat calls, and 1,939 as *Myotis* calls. In 2016, 3,270 bat passes were classified as Low bat calls, 929 as Mid bat calls, and 584 as *Myotis* calls.

### 3.1 Evidence for spatial partitioning

**FIGURE 2:**
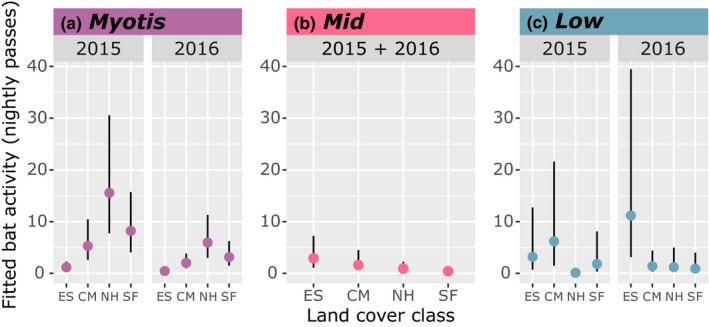
Relationships between land cover class and bat phonic group activity in Great Smoky Mountains National Park, USA. Land cover classes were conifer‐mixed hardwood (CM), northern hardwood (NH), spruce‐fir (SF), and early successional (ES) classes, and phonic groups were a) *Myotis*, b) Mid, and c) Low. Differences in activity levels across land cover classes are presented as fitted means (calculated from the full model while holding distance from water, relative humidity, and temperature at mean values) with bars representing the 95% confidence interval (calculated using bootstrap resampling with 1,000 simulations)

**FIGURE 3:**
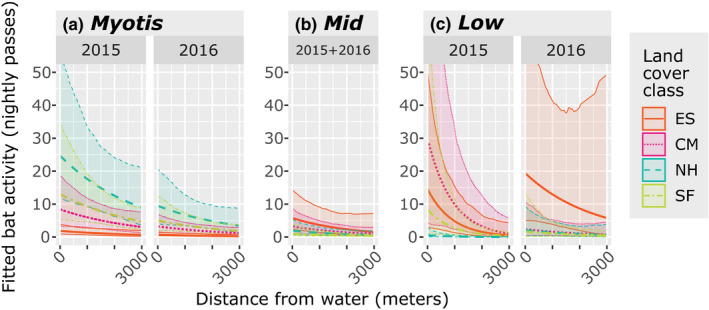
Fitted relationships from mixed‐effects models relating activity of three bat phonic groups (a) *Myotis*, (b) Mid, and (c) Low to distance from water in Great Smoky Mountains National Park, USA. Fitted values were estimated from the full model while holding other covariates (relative humidity and temperature) at mean values. The colored shading represents the 95% confidence intervals, estimated from bootstrap resampling with 1,000 simulations. Upper confidence intervals for Low bats in CM (2015), SF (2015), and ES (2016) have been clipped to better visualize relationships. Upper confidence interval predictions reached 140 calls in CM and 90 in SF at sites adjacent to water in 2015 and reached 75 in ES at sites adjacent to water in 2016

Year, land cover class, and proximity to water were important predictors of *Myotis* activity. The most plausible model for *Myotis* included year without interactions with distance to water or land cover class. From 2015 to 2016, *Myotis* activity declined by 70% (*z* = −4.93, *p* = 8.13e‐07), but overall patterns of spatial resource use remained consistent (Figure 2a). *Myotis* activity was the greatest overall in northern hardwood forests and the lowest in early successional openings (Figure 2a). *Myotis* activity was 62% greater in mid‐elevation northern hardwood forests than in high‐elevation spruce‐fir forests, 43% greater in spruce‐fir forests than in low‐elevation conifer‐mixed hardwood forests, and > 100% greater in conifer‐mixed hardwood forests than in early successional openings (128% greater in 2015 and 163% greater in 2016; Figure 2a). Holding other variables at their means, *Myotis* activity was up to 3 times lower at sites 3,000 m from water versus sites 0 m from water (Figure 3a).

Despite their similarities—larger size and lower predicted clutter tolerance—the Low and Mid bats used space differently. Neither year, land cover class, nor proximity to water were important predictors of Mid bat activity, as none of the models outperformed the null model (Figures 2b and 3b). However, year, land cover class, and proximity to water were important predictors of Low bat activity. The most plausible model for Low bats included year as an interactive effect with land cover class and water availability. Depending on the year, Low bat activity was the greatest overall in either conifer‐mixed hardwood forests or early successional openings (Figure 2c). Low bat activity was lowest in either northern hardwood or spruce‐fir forests (Figure 2c). In 2015, Low bat activity was 64% greater in conifer‐mixed hardwood forests than in early successional openings, 54% greater in early successional openings than in spruce‐fir forests, and 177% greater in spruce‐fir forests than northern hardwood forests (Figure 2c). In 2016, Low bats shifted to using early successional openings more than the forested land cover classes. Low bats were 156% more active in early successional openings than conifer‐mixed hardwood forests, 15% more active in conifer‐mixed hardwood forests than northern hardwood forests, and 26% more active in northern hardwood forests than spruce‐fir forests (Figure 2c). Holding other variables at their means, Low bat activity was up to 14 times lower at sites 3,000 m from water versus sites 0 m from water; proximity to water was a better predictor of Low bat activity in 2015 versus 2016 (Figure 3c).

### 3.2 Evidence for temporal partitioning

**FIGURE 4:**
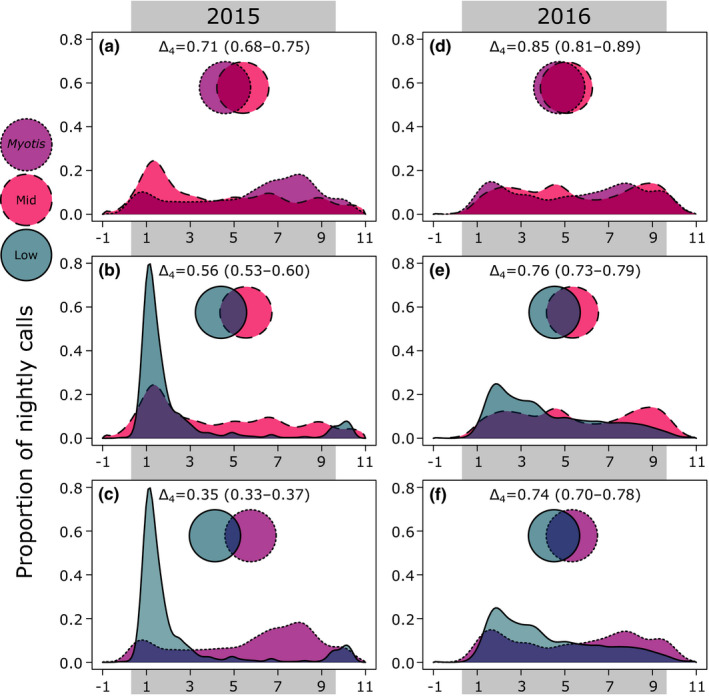
The degree of nightly temporal overlap between *Myotis* and Mid (a,d), Mid and Low (b,e), and *Myotis* and Low (c,f) bat phonic groups in 2015 (a,b,c) and 2016 (d,e,f) in Great Smoky Mountains National Park, USA. Models were generated using a kernel density estimation procedure yielding an overlap value, Δ_4_, indicative of the mutual area under the two presented curves. The Δ_4_ value ranges from 0 (no temporal overlap) to 1 (complete temporal overlap) and is followed by a bootstrapped confidence interval in parentheses (based on 1,000 simulations)

Temporal patterns in phonic group activity varied across the night and years, with differential activity peaks and troughs driving variation in the degree of temporal overlap between bat phonic groups (Figure 4). In 2015, the greatest overlap in temporal use was between Mid and *Myotis* bats (Δ_4_ = 0.71, Figure 4a), while Low bats were comparatively distinct in terms of their activity patterns. Across both years, Low bat activity patterns were more like patterns of Mid bats (Δ_4_ = 0.56, Figure 4b) than *Myotis* (Δ_4_ = 0.35, Figure 4c). In contrast to the other two phonic groups, Low bats showed strongly unimodal activity patterns, especially in 2015 when Low bat activity peaked 2–3 hr after sunset, declining to nearly no activity by the fourth hour of the night. Low bats remained largely absent from the landscape until a small peak just before dawn (Figure 4b or c). However, Mid and *Myotis* were present on the landscape throughout the night, with small peaks in *Myotis* activity 1–2 hr after sunset, large peaks in Mid activity 2–3 hr after sunset, and large peaks in *Myotis* activity 9 hr after sunset (Figure 4a). The primary activity peak for Mid bats (peak of greatest activity) corresponded to the primary activity peak for Low bats (Figure 4b), but the primary activity peak for *Myotis* occurred when Low bats were the least active (Figure 4c) and Mid bat activity was reduced (Figure 4a).

All phonic groups shifted their mean nightly activity patterns from 2015 to 2016, but not to the same extent. We observed the greatest activity shift in Low bats, followed by Mid, and then *Myotis* bats. Broadly, the degree of temporal overlap between each phonic group pair was greater in 2016 (Figure 4). In 2016, the primary Low bat activity peak still occurred 2–3 hr after sunset, but it was much less dramatic, and Low bats remained relatively active throughout the night (Figure 4e,f). In 2016, there was no discernible primary peak in Mid bat activity, although activity pulsed 3, 6, and 10 hr after sunset. This final pulse corresponded with a period of reduced Low bat activity (Figure 4e). *Myotis* also exhibited no discernible primary peak in activity, but activity pulsed approximately 2, 9, and 11 hr after sunset (Figure 4d,f). These pulses were slightly offset from pulses in Mid bat activity (Figure 4d), and the pulses around 9 and 11 hr after sunset corresponded with periods of declining Low bat activity (Figure 4f).

We observed greater similarity in the activity patterns of Mid and *Myotis* than between either group and Low bats, a trend that was consistent from 2015 to 2016. Although the degree of temporal overlap between all phonic groups increased in 2016, the overall pattern of greater temporal overlap between Mid and *Myotis* was conserved (Δ_4_ = 0.71 in 2015, Δ_4_ = 0.84 in 2016; Figure 4a,d). Low bats were comparatively isolated in their activity patterns both years (Figure 4). There was no statistical difference between the degree of overlap between Low and Mid (Δ_4_ 0.76, Figure 4e) or Low and *Myotis* (Δ_4_ = 0.74, Figure 4f) in 2016.

**FIGURE 5:**
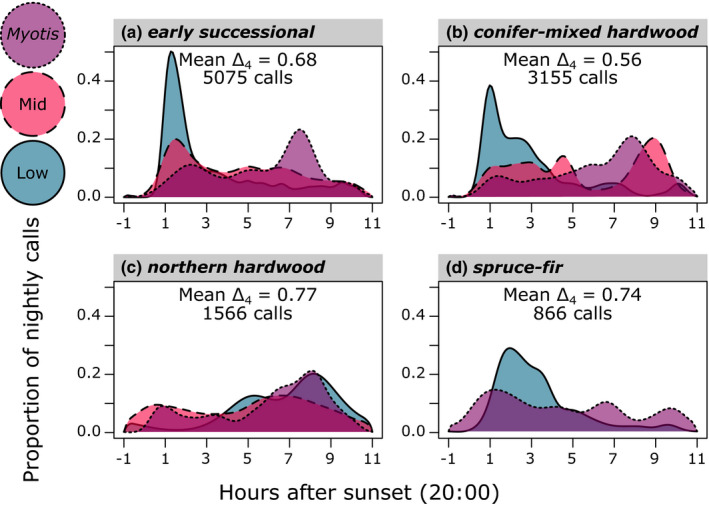
The degree of nightly temporal overlap between Low (blue, solid line), Mid (red, dashed line), and *Myotis* (purple, dotted line) phonic groups in the four dominant land cover classes of Great Smoky Mountains National Park, USA. Models were generated using a kernel density estimation procedure yielding an overlap value, Δ_4_, indicative of the mutual area under the two presented curves. The Δ_4_ value ranges from 0 (no temporal overlap) to 1 (complete temporal overlap). We omitted mid bat activity from panel d due to its low activity in this land cover class

Patterns of temporal overlap among phonic groups varied by land cover class (Figure 5). Phonic groups were the most temporally separated in their activity levels in the conifer‐mixed hardwood forests (Δ_4_ = 0.56, Figure 5b), followed by early successional openings (Δ_4_ = 0.68, Figure 5a), spruce‐fir forests (Δ_4_ = 0.74, Figure 5d), and northern hardwood forests (Δ_4_ = 0.77, Figure 5c).

## 4 DISCUSSION

**FIGURE 6:**
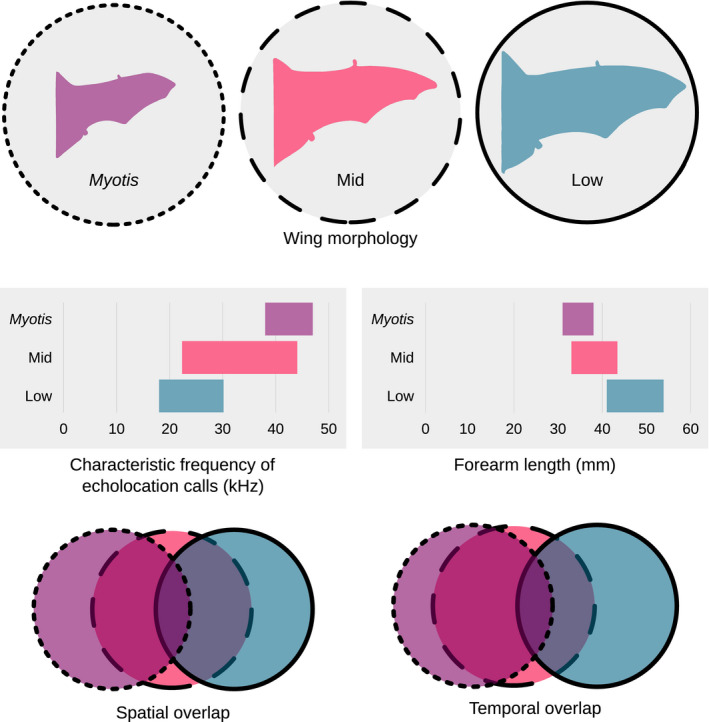
The ecomorphology and spatiotemporal separation of *Myotis*, Mid, and Low bat phonic groups in this study. *Myotis leibii*, *Lasiurus borealis*, and *Eptesicus fuscus* were used for the *Myotis*, Mid, and Low bat wing silhouettes, respectively, inspired by Farney and Fleharty (1969). The characteristic frequencies and forearm lengths represent a range of means for the bat species contained within each phonic group. Characteristic frequencies were informed by Szewczak (2013), and forearm lengths were produced from our capture database (O'Keefe et al., 2019). Spatial and temporal overlap charts were generated from the results of this study

In a diverse and heterogeneous landscape, bat activity varied in both space and time in ways consistent with the prediction that diverse assemblages should partition resources along multiple resource axes to coexist. *Myotis* and Low bats were the most spatially and temporally dissimilar, while Mid bats employed a more generalist spatiotemporal strategy (Figure 6). Phonic groups changed their temporal and, to a lesser extent, spatial resource use across the 2‐year study. Notably, we found evidence for relaxation in the temporal niches of all phonic groups from 2015 to 2016. The degree of temporal separation between bat phonic groups varied across the landscape—temporal partitioning was more apparent in the conifer‐mixed hardwood forests and early successional openings, which also saw greater overall bat activity. Overall, these findings support the hypothesis that niche separation in predator assemblages may occur along multiple resource axes, adding to the growing body of evidence that bats partition their temporal activity, and reinforcing the notion that temporal partitioning affords bats the flexibility to respond to environmental cues.

Low bats used low‐elevation conifer‐mixed hardwood forests and early successional openings near water, consistent with expectations based on ecomorphology and previous research. Many bat species in the Low phonic group are predicted to have lower maneuverability in cluttered environments (Aldridge & Rautenbach, 1987; Norberg & Rayner, 1987), implying increased foraging efficiency in open or sparsely vegetated sites. This is congruent with our finding that Low bats were more active in early successional openings and conifer‐mixed hardwood forests, which have more vertical space and are less cluttered than the northern hardwood forests and spruce‐fir forests. *Eptesicus fuscus* (the most commonly captured Low bats at our study site) are often associated with uncluttered sites (Brooks et al., 2017; Johnson et al., 2010; Loeb & O'Keefe, 2006). Low bats may have foraged above the canopy in any of the forested land cover classes, out of range of detection by our microphones. For example, *E. fuscus*, *N. humeralis*, and *L. cinereus* are more frequently recorded above the forest canopy than within or below it in the Coastal Plain of South Carolina (Menzel, Menzel, et al., 2005), while *L. cinereus* and *Myotis*, but not *E. fuscus*, partition vertically in the boreal forests of Canada (Kalcounis et al., 1999). The prevalence of vertical partitioning likely depends on a variety of local conditions, including forest structure and prey availability.

Land cover class and water availability were not strong predictors of Mid bat activity in this landscape, which may result from the inclusion of a common generalist bat, *L. borealis,* the eastern red bat, in this phonic group or from limitations in our study design. *L*. *borealis* do not exhibit clear patterns of landscape‐level habitat selection (Carter, 1998; Elmore et al., 2005), except to avoid urban development (Walters et al., 2007). Where evidence of selection is present, *L. borealis* select for linear features that facilitate flight, including roads, ridgetops, and streams (Amelon et al., 2014), rather than specific land cover classes. Though our findings agree with the general trends of published literature, they may be biased because we deployed our detectors on trails. Alternatively, any evidence of spatial partitioning within the Mid bat group may be obscured by the fact that some of the bats within the Mid bat phonic group are ecologically dissimilar (Table 1).

*Myotis* were comparatively distinct in their spatial activity patterns, avoiding early successional openings in favor of all the forested land cover classes. The apparent preference of *Myotis* for forested land cover classes matches expectations in light of their ecomorphology—short, broad wings, low wing loading, and broadband, high‐frequency calls (Aldridge & Rautenbach, 1987; Norberg & Rayner, 1987). Several *Myotis* species that occur in our study area exhibit strong selection for forested land cover (e.g., *M. leibii*: Johnson et al., 2009; *M. septentrionalis*: Henderson & Broders, 2008; *M. sodalis*: Menzel, Ford, et al., 2005; Sparks et al., 2005) and respond negatively to fragmentation or loss of forests (*M. septentrionalis*: Henderson et al., 2008). However, the use of openings by *Myotis* species is less clear, with *Myotis* avoiding them in some landscapes but selecting for them in others. *Myotis* activity is often greater in intact forest patches than clear‐cuts (Owen et al., 2004; Patriquin & Barclay, 2003) or forest edges (Morris et al., 2010), and there is evidence that *M. sodalis* avoid agricultural fields and pastures when commuting from roosting to foraging habitat (Murray & Kurta, 2004). However, small natural gaps may provide important foraging opportunities for *Myotis*, particularly within densely stocked or regenerating forests. For example, *M. septentrionalis* are more likely to be recorded at sites with low or medium density vegetation than sites with dense vegetation (Loeb & O'Keefe, 2006).

The importance of gaps or openings to *Myotis* may vary given the context of the larger landscape matrix (Loeb & O’Keefe, 2011) and the characteristics of the opening (Brooks et al., 2017). Because clutter‐adapted *Myotis* are not restricted to foraging in complex or cluttered habitats, we might expect *Myotis* to exhibit some flexibility in their foraging strategy, moving into early successional openings to take advantage of resource pulses in an environment where flight costs should be comparatively low. However, our data suggest this may be a rare occurrence in this landscape. Insect productivity is 2.5 times higher in the forests than the grassy balds of this landscape (Whittaker, 1952) so forested sites may, on average, provide more insect‐rich foraging opportunities than early successional openings. Insect productivity is also 1.4 times greater in mid‐ versus low‐elevation forests and 2 times greater in high‐ versus low‐elevation forests (Whittaker, 1952), suggesting the mid‐elevation northern hardwoods and high‐elevation spruce‐fir forests may indeed be quality foraging habitats for bats that can effectively forage in these more cluttered forest types. *Myotis*, being adept at flying in clutter, may preferentially exploit these accessible and insect‐rich forests with little need for early successional openings. Conversely, the less clutter‐adapted Low bats may be biomechanically constrained from efficiently foraging in the cluttered, high‐elevation forest types (Mayberry et al., 2020). In 2016, we netted 3 spruce‐fir sites to validate the initial observation of high *Myotis* activity in that land cover class and captured several *Myotis leibii* (J. M. O’Keefe, E.A. Beilke unpublished data). This suggests *M. leibii* may drive the *Myotis* activity patterns we observed in spruce‐fir forests in this study.

Temporal partitioning may facilitate the coexistence of bats in this landscape, particularly where bats overlap spatially. Phonic groups were the most temporally isolated from each other in the conifer‐mixed hardwood forests and the early successional openings, which also saw the highest overall levels of bat activity (Figure 5a,b). Temporal segregation was comparatively low in northern hardwood and spruce‐fir forests, which saw low overall bat activity (Figure 5c,d). These observations support the hypothesis that temporal partitioning may be a flexible strategy employed to minimize interspecific competition in more crowded environments (Adams & Thibault, 2006; Razgour et al., 2011), though it bears mentioning that the ubiquity of interspecific competition in bat assemblages is unclear (Salinas‐Ramos et al., 2020). The temporal separation we observed could instead reflect the variable activity patterns of the preferred prey of each phonic group (Rydell et al., 1996). For example, low‐frequency bats prey more heavily on beetles (e.g., Clare et al., 2014b; Wray et al., 2021), while *Myotis* mainly depredate small‐bodied moths and flies (e.g., Clare et al., 2014a; O'Rourke et al., 2021).

The prevalence of temporal partitioning in bat assemblages could easily be underestimated if spatiotemporal context is not considered. Overall bat activity peaked just after sunset and each phonic group was active during this period; however, pulses of activity by phonic group were more segregated after this initial activity period. Consequently, partial‐night surveys may be insufficient to study partitioning in bat assemblages. Additionally, temporal partitioning was inconsistent across years, land cover classes, and phonic group pairs, emphasizing the importance of scope in temporal partitioning studies; by narrowing the scope of a study to a few nights, sites, or species, we would likely miss temporal partitioning. These factors may explain the lack of temporal partitioning in several studies (e.g., Adams & Fenton, 2017; Arlettaz, 1999; Fenton & Rautenbach, 1987). However, scope is not enough, as we might wrongfully conclude from our dataset that this assemblage does not partition their temporal activity had we simply quantified the degree of overlap across all years and land cover classes, as the average degree of overlap was relatively high (65%). Context was important too, as temporal partitioning was more dramatic in 2015 than in 2016 and certain land cover classes. The variable and contextual nature of temporal partitioning in bat assemblages may provide an excellent system for studying the conditions and mechanisms that lead to temporal partitioning in species assemblages.

Temporal segregation, which relaxed from 2015 to 2016, was more flexible than spatial segregation which may be due to a range of dynamic processes. We may have observed niche relaxation associated with a change in the competitive landscape from 2015 to 2016, such as might be caused by the decline of one or more species from an assemblage. For example, there is evidence that the spatiotemporal (Jachowski et al., 2014; Mayberry et al., 2020; Teets, 2018) and dietary (Morningstar et al., 2019) niches of bats unaffected by WNS have relaxed in WNS‐positive landscapes, presumably due to the decline of *Myotis* in those assemblages. Due to WNS, *Myotis* have been declining in the Great Smoky Mountains landscape since at least 2014 (O'Keefe et al., 2019), and we recorded 70% fewer *Myotis* calls than in 2016 than in 2015. Our findings agree with previous reports of niche‐relaxation co‐occurring with *Myotis* spp. declines. However, the broad observation that bats are expanding their niches through time might be related to other extrinsic factors. For example, because temporal partitioning in predators is inherently tied to the activity patterns of their prey (Kronfeld‐Schor & Dayan, 2003; Schoener, 1974), and given the mounting evidence that insects are declining (Wagner, 2020), bats may be expanding their niches through time in response to declines in prey availability. Either scenario is outside the scope of our 2‐year study. It is equally possible these shifts were driven by annual fluctuations in weather or insect availability.

This work has important implications for how we survey bat populations—namely that partial‐night surveys may not capture a representative sample of the local bat assemblage. It is a common practice in mist‐net and driving transect surveys to exclusively sample the first 1–5 hr following sunset (Loeb et al., 2015; U.S. Fish & Wildlife Service, 2020). However, if *Myotis* are more active in the latter part of the night, then these approaches may miss the majority of *Myotis* activity (Johnson et al., 2011).

Temporal partitioning among bats may be more common than previously thought, and this phenomenon is worth exploring in other settings, ideally with more sampling locations and over a longer period. Multi‐night, passive acoustic studies are relatively easy to implement and lend themselves to studying spatiotemporal trends in activity (Frick, 2013; Obrist, 2020). We recommend researchers use their acoustic datasets to examine patterns of spatiotemporal resource use to determine how widespread temporal segregation is and to describe its role in structuring bat assemblages. If temporal partitioning is revealed to be widespread among bats, they may serve as model organisms for studying temporal partitioning—its importance relative to other forms of partitioning, and the conditions that spur temporal segregation (Kronfeld‐Schor & Dayan, 2003).

## CONFLICT OF INTEREST

None declared.

## AUTHOR CONTRIBUTIONS

**Elizabeth A. Beilke:** Data curation (lead); Formal analysis (equal); Investigation (lead); Methodology (equal); Visualization (lead); Writing‐original draft (lead); Writing‐review & editing (equal). **Rachel V. Blakey:** Conceptualization (equal); Formal analysis (equal); Methodology (equal); Writing‐review & editing (equal). **Joy M. O'Keefe:** Conceptualization (equal); Funding acquisition (lead); Investigation (supporting); Methodology (equal); Project administration (lead); Resources (lead); Writing‐review & editing (equal).

## Data Availability

The data that support the findings of this study are openly available in Dryad (https://doi.org/10.5061/dryad.66t1g1k1t).
